# Source-Specific PM_2.5_ Exposure and Associated Health Risks During Beijing Winter

**DOI:** 10.3390/toxics13121081

**Published:** 2025-12-16

**Authors:** Xin Liu, Zhiqing Liu, Wenming Pei, Xiaoyu Zhang, Xiaoting Jie, Zhi Yang, Liwei Liu, Yuxing Gao, Ruoyu Hu, Mingzhu Zhang

**Affiliations:** 1 Nanjing Institute of Environmental Sciences, Ministry of Ecology and Environment of China, Nanjing 210042, Chinapeiwenming@nies.org (W.P.);; 2 Dali Ecological Environment Monitoring Station of Yunnan Ecological Environment Department, Dali 671000, China; 3 Lintong District Meteorological Bureau of Xi’an City, Xi’an 710600, China; 4 Xi’an Meteorology Bureau of Shaanxi Province, Xi’an 710016, China

**Keywords:** PM_2.5_, human health, AF, Ens-HYSPLIT-CWT

## Abstract

Atmospheric fine particles (PM_2.5_, aerodynamic diameter ≤ 2.5 µm) have a serious effect on human health. This study combined concentration weighted trajectory (CWT) analysis with the HYSPLIT trajectory ensemble (Ens-HYSPLIT-CWT), to separate the sources of PM_2.5_ transported to Beijing, and further investigate the effect of PM_2.5_ originated from different sources on human health. We found that north region air masses usually come with clean events under the blessing of meteorological conditions, combined with the clean air mass transported from the north, as high wind speed near the surface promotes the horizontal diffusion of pollutants. Additionally, north region air masses contribute to the decrease in aerosol optical depth (AOD) at Beijing and surrounding areas, with AF (daily attributable fraction associated with short-term PM_2.5_ exposure) values of Beijing only at 0.14. During the study period (from January to March 2024), south region air masses usually come with high PM_2.5_ values, which is correlated to the meteorological conditions and pollutant spatial distribution. The air masses coming from the south region contain high temperature and relative humidity (RH), promoting the occurrence of high pollution events. AOD spatial distribution observed from satellites showed that except for the dominance of north region air mass sources, the south region presents high AOD values, further resulting in the highest AF value of 0.75 obtained at Beijing, which is 5 times higher than the north region’s dominant AF mean value. It is worth noting that the air mass originated from the east region, which originally contributed relatively clean air masses before emission reduction, increased its contribution to air mass pollution after emission reduction due to the decrease in pollution concentration in other regions. As a result, the mean PM_2.5_ in this source area was second only to south region air masses and local emission sources, and the AF value even exceeded local emissions, second only to south region air mass sources, reaching 0.5. This result emphasizes that in future pollution control policy adjustments and research on human health, attention needs to be paid to the contribution of eastward air masses.

## 1. Introduction

Atmospheric fine particles (PM_2.5_, aerodynamic diameter ≤ 2.5 µm) poses significant effects on climate systems [1] and human health [2,3,4]. Studies have shown that exposure to PM_2.5_ increases the risk of respiratory and cardiovascular diseases [5,6,7,8,9,10]. The rapid industrialization and urbanization in China over recent decades has resulted in severe air quality deterioration, particularly in urban areas [11]. Fortunately, regulatory measures such as the Air Pollution Prevention and Control Action Plan (2013–2017) have successfully reduced anthropogenic emissions and ambient PM_2.5_ [12,13]. However, despite the decrease of PM_2.5_ concentration, recent epidemiological assessments estimate that PM_2.5_ exposure was associated with approximately one million premature deaths in China during 2017 [14].

Recent advances in health science have enhanced our understanding of the effects of respirable particles on human health. The correlation between fine particle exposure and human mortality was first conclusively demonstrated by a seminal Harvard study across six American cities in 1996. This study established that PM_2.5_ significantly increased non-accidental death rates, particularly among the elderly (RR = 1.5%, 95% CI: 1.1–1.9%) [15]. Epidemiological evidence demonstrates a direct correlation between PM_2.5_ exposure and excess mortality, with respiratory disease patients being particularly vulnerable to the effects of air pollution. A substantial portion of non-accidental deaths has been linked to poor air quality, especially in regions with elevated particle concentrations. Studies indicate that PM_2.5_ associated excess deaths in China reached 1.3 million in 2013 [16]. A comprehensive 25-year analysis of global disease burden by Cohen et al. [17] revealed that ambient PM_2.5_ was linked to 1.1 million premature deaths across the country in 2015. Further research documented approximately 170,000 additional deaths from acute PM_2.5_ exposure in 2015 [18], while the combined mortality impact from all air pollutants was estimated at 1.35 million deaths in 2017 [19]. Recent assessments by Lelieveld et al. [20] indicate that outdoor air pollution contributed to approximately 1.36 million premature deaths across China in 2020, while multiple epidemiological studies have quantified the mortality burden from fine particle exposure in the country.

Back-trajectory analysis is often employed in air mass source attribution studies. The HYSPLIT model serves as a key tool for tracking air parcel pathways and identifying pollution origins. For instance, Wu et al. [21] recently applied HYSPLIT in Zhoushan to analysis 24 h backward trajectories, classifying air masses as continental when their pathways spent over 5% of the previous day above land masses, while designating remaining trajectories as marine-influenced. Statistical clustering techniques are frequently employed to categorize multiple air mass trajectories over extended monitoring periods. Li et al. [22] employed cluster analysis in Wuhan to identify distinct air mass patterns during autumn 2019, revealing three primary transport pathways: local circulation, northerly flows, and long-range transport from northeastern and coastal regions. However, clustering approaches have inherent limitations: the algorithms may separate trajectories with similar directional origins into different clusters based solely on transport distance, potentially complicating source attribution.

The Chinese government has implemented two emission reduction measures (the Air Pollution Prevention and Control Action Plan from 2013 to 2017 and the Blue-Sky Protection Campaign from 2018 to 2020), effectively reducing PM_2.5_ concentrations and weakening their impact on human health during polluted events. The impact of PM_2.5_ on human health before the emission reduction, i.e., before 2020, is no longer applicable to the current environment and conditions. During the COVID-19 pandemic (December 2019 to December 2022), the government controlled a significant reduction in anthropogenic emission sources, resulting in PM_2.5_ concentrations that are not representative of actual emissions. Model simulations can quantify PM_2.5_ concentration from different sources but may not fully and effectively reproduce observed PM_2.5_ concentrations. Researchers commonly use the HYSPLIT backward trajectory model to distinguish the main sources of polluted air masses when using only observation data. However, traditional approaches combining HYSPLIT back-trajectories with cluster analysis have limitations in accurately characterizing pollution transport pathways [21]. Air masses with the same direction but different transmission distances will be divided into different sources, resulting in errors in air mass source classification, thus affecting the assessment of the impact of air masses from different sources on human health. To address these methodological constraints, we present an enhanced analytical framework that integrates concentration-weighted trajectory (CWT) analysis with HYSPLIT ensemble trajectories (Ens-HYSPLIT-CWT). This combined approach enables more robust differentiation of air mass origins and provides deeper insights into the health burden associated with PM_2.5_ from various source regions, especially under the current background of emission reductions.

## 2. Materials and Methods

**Figure 1:**
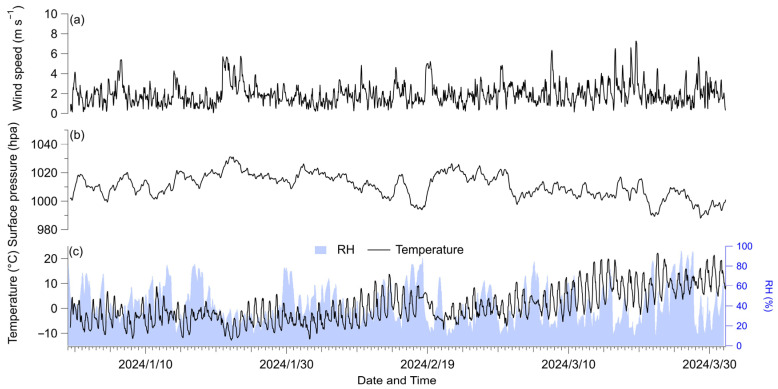
Temporal evolution for wind speed (**a**); surface pressure (**b**); and temperature and relative humility (RH) (**c**).

**Figure 2:**
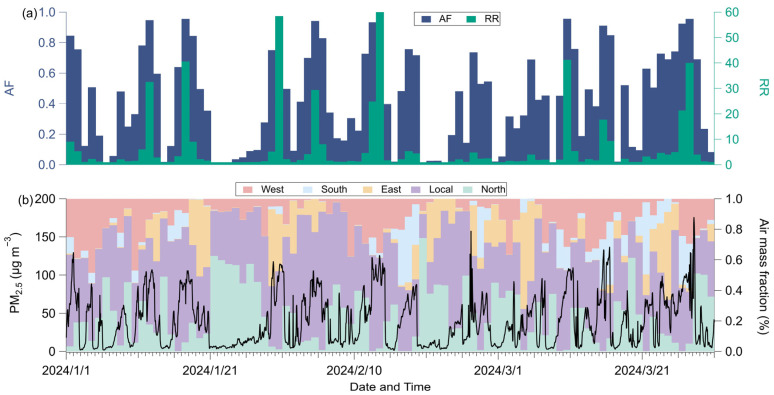
Temporal evolution for (**a**) daily attributable fraction (AF) and relative risk (RR) associated with short-term exposure to PM_2.5_; (**b**) PM_2.5_ and air mass fraction derived from the Concentration-Weighted Trajectory (CWT) method (**b**).

Meteorological data, including temperature, relative humidity (RH), surface pressure, and wind speed (Figure 1), were obtained for the latitude and longitude coordinates of ground observation stations from the European Centre for Medium-Range Weather Forecasts (ECMWF) ERA5 reanalysis dataset, available at https://cds.climate.copernicus.eu/datasets (accessed on 1 December 2025). The PM_2.5_ data (Figure 2b) were obtained from in situ air quality monitoring conducted by the China National Environmental Monitoring Center, with the monitoring station located in Chaoyang (39.98° N, 116.40° E), situated in the central urban area of Beijing (from January to March 2024).

### 2.1. Satellite Data

Our analysis of atmospheric aerosol properties utilized Collection 6 (C006) Level 3 aerosol optical depth (AOD) measurements at 550 nm wavelength, integrating retrievals from both Dark Target (DT) and Deep Blue (DB) algorithms. These observations were obtained through the Moderate Resolution Imaging Spectroradiometer (MODIS) aboard NASA’s Aqua platform. The MODIS instrument series, deployed on both Terra and Aqua satellites, represents NASA Goddard’s advanced Earth observation system, with Terra crossing the equator during morning hours (10:30 LT, descending node) and Aqua during afternoon periods (13:30 LT, ascending node) [23,24]. The MODIS sensor system achieves comprehensive global coverage through its extensive viewing swath (~2330 km) and frequent revisit capability (1–2 days), collecting data across 36 spectral channels. Aerosol retrievals specifically utilize seven specialized bands spanning near-UV to near-IR wavelengths (0.415–2.155 μm), enabling sophisticated AOD determinations over diverse surface types [23,25]. The retrieval methodology implements distinct algorithmic approaches: the DT algorithm suite processes vegetated and dark-soil regions, with separate variants optimized for terrestrial and oceanic surfaces [26,27], while the enhanced DB algorithm, refined in C006, extends coverage to bright surfaces and provides comprehensive land surface capabilities [28]. All satellite data products were accessed and processed through NASA’s Giovanni web interface (https://giovanni.gsfc.nasa.gov/giovanni/, accessed on 1 December 2025), facilitating standardized data acquisition and analysis protocols.

### 2.2. Air Mass Sources

**Figure 3:**
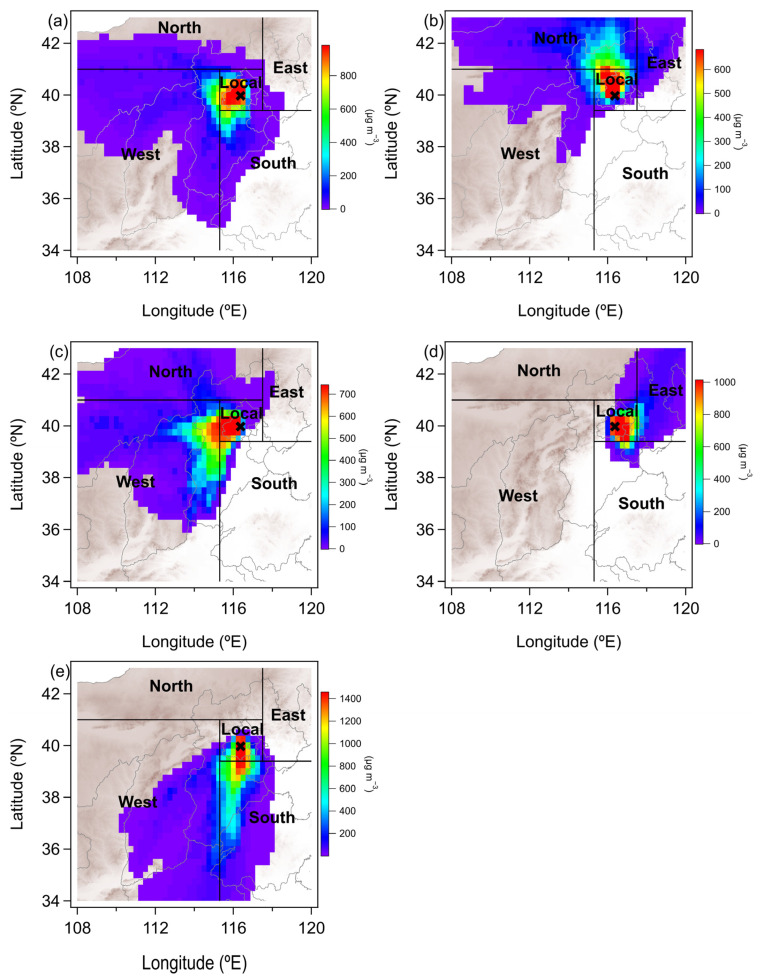
Typical examples of CWT analysis are shown for (**a**) a local emission event (8 January 2024); (**b**) a north region transport event (9 January 2024); (**c**) a west region transport event (10 January 2024); (**d**) an east region transport event (19 January 2024); (**e**) a south region transport event (17 February 2024).

Source attribution (Figure 3) was performed using the Ens-HYSPLIT-CWT method, following recent developments by Hu et al. [13]. Hu et al.’s method improved upon traditional CWT analysis, which cannot provide hourly resolution for pollutant source analysis, enabling the use of CWT to analyze pollutant sources at an hourly resolution. Back-trajectories were computed at three-hour intervals using meteorological inputs from GDAS reanalysis (1-degree resolution, available at ftp://arlftp.arlhq.noaa.gov/pub/archives/gdas1, accessed on 1 December 2025). For each analysis time, the model generated 27 ensemble members tracking 24 h air parcel pathways ending at our Beijing monitoring site (39.98° N, 116.40° E, 250 m a.s.l.).

The CWT methodology assigns pollution source intensities by integrating air mass residence times with measured pollutant concentrations across a 0.25-degree spatial grid. This statistical approach computes weighted average concentrations for each grid cell traversed by back-trajectories, providing quantitative estimates of regional source contributions to pollution levels at the receptor site [29,30,31].

(1) $$C_{ij}=\frac{\sum_{l=1}^{M}C_{l}\times \tau_{ijl}}{\sum_{l=1}^{M}\tau_{ijl}}\times W\left(n_{ij}\right)\;$$

(2) $$W\left(n_{i,j}\right)=\left\{\begin{array}{l} 1.00,\;3n_{ave}<n_{ij}\\ 0.70,\;1.5n_{ave}<n_{ij}\leq 3n_{ave}\\ 0.42,\;n_{ave}<n_{ij}\leq 1.5n_{ave}\\ 0.05,\;n_{ij}\leq n_{ave} \end{array}\right.$$

The grid-specific weighted concentration $C_{ij}$ integrates the average weighted concentration, where individual trajectories *l* contributes to a total of *M* pathways. $C_{l}$ is the PM_2.5_ concentration measured at the receptor site. The residence time parameter $\tau_{ijl}$ quantifies how long each trajectory remains within a specific grid cell. In calculation, trajectory frequency per grid cell replaces absolute residence time calculations. Additionally, a weighting function *W*(*n_i,j_*) is applied to reduce uncertainties in cells with limited trajectory counts, where $n_{ij}$ denotes trajectory frequency per grid cell and $n_{ave}$ represents the mean trajectory count across all cells.

Source attribution was accomplished through CWT analysis of 648 daily trajectories, with the study domain subdivided into five geographical sectors: the central Beijing region (115.3–117.5° E, 39.4–41° N), western plateau region (108–115.3° E, 34–41° N), northern plateau area (108–117.5° E, 41–43° N), eastern coastal plain (117.5–120° E, 39.4–43° N), and southern region (115.3–120° E, 34–39.4° N). Regional contributions were quantified by integrating CWT-derived concentrations across grid points within each defined sector (Figure 2b).

### 2.3. Health Burden Assessment

The estimated health burden owing to short-term PM_2.5_ exposure can be calculated as follows [32,33,34]:

(3) $$RR=\exp\left(\beta \times \left(C-C_{0}\right)\right)$$

(4) $$AF=\left(RR-1\right)/RR$$

were RR indicates the daily relative risk associated with short-term PM_2.5_ exposure, β is the concentration-response coefficient for health endpoints exposed to PM_2.5_, ranging from 0.31 to 0.45, with 0.38 used in this manuscript as suggested by recent epidemiological studies [35,36]. The influence of *AF* on the results is less than 16% when using the maximum and minimum values of β. C is the daily PM_2.5_ concentration, and $\;C_{0}$ is the daily PM_2.5_ threshold concentration, which is assumed to be zero, similar to previous studies [19,37]. AF indicates the daily attributable fraction associated with short-term PM_2.5_ exposure.

## 3. Results and Discussion

### 3.1. Meteorology Influenced by Diverse Air Mass Sources

**Figure 4:**
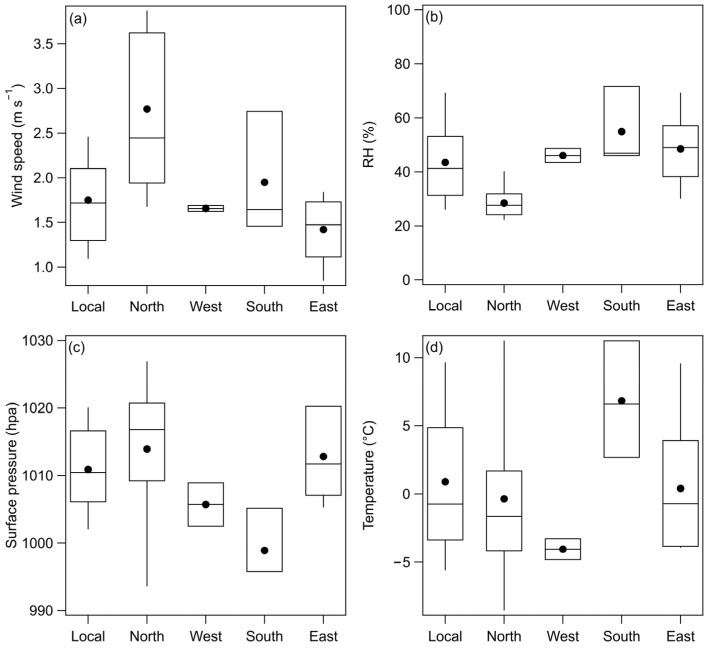
Wind speed (**a**); RH (**b**); surface pressure (**c**) and temperature (**d**) distribution dominated by different air masse sources. Solid circle indicates mean value, upper and lower boundaries represent 75th and 25th percentiles, and whiskers above and below the box are 90th and 10th percentiles.

We conducted a statistical analysis of the meteorological conditions in the Beijing area under the influence of different air mass sources during the research period (Figure 4). The average wind speed in Beijing was highest (3 m s^−1^) when air masses originated from the north region, approximately twice as high as the lowest average wind speed (1.4 m s^−1^) associated with air masses from the east region. The 75th percentile wind speed for north region air masses was 3.6 m s^−1^, the highest among all air mass sources, while the 25th percentile wind speed (1.9 m s^−1^) exceeded the 75th percentile wind speed of both eastward and westward air masses. Average wind speeds during periods influenced by west region air masses and local emissions were similar at 1.7 m s^−1^. South region air masses were associated with an average wind speed of 1.9 m s^−1^ in the Beijing area. Surface wind speed can impact pollutant transport and diffusion, low wind speeds reduce the horizontal diffusion of pollutants, potentially exacerbating pollution concentrations and posing a threat to human health.

RH is another important factor affecting polluted events. High RH can lead to increased moisture absorption by particulate matter, resulting in an increase in particle size and further affecting optical properties, such as visibility, thereby exacerbating pollution events. As shown in Figure 4b, both the mean and distribution results indicate that air masses originating from the south region have the highest RH among all incoming air masses, with a mean of 55%, a 75th percentile value of 72%, and a 25th percentile value of 46%. This is closely related to the climate differences between northern and southern China, with more warm and humid air masses in the south and more dry and cold air masses in the north. Therefore, under the influence of southward air masses, warm and humid air moves into the Beijing area, leading to increased RH. In contrast, the lowest RH values in Beijing are associated with air masses from the north region, with an average of only 28%. Apart from the south region air masses, the second-highest RH is contributed by east region air masses (49%), followed by westward air masses (46%) and locally emitted air masses (43%).

Regarding temperature, the Beijing area exhibits the highest average temperature (6.8 °C) when under the influence of south region air masses. This is directly related to the latitudinal temperature variation, as the southern region is closer to the point of direct sunlight and experiences higher temperatures. The movement of warm air masses from the south to the Beijing area increases the temperature. The lowest average temperature (−4 °C) occurs under the influence of west region air masses, followed by north region air masses (0 °C) and local emission (1 °C). In terms of surface pressure, the Beijing area experiences the lowest average pressure (999 hPa) when influenced by south region air masses, followed by west region air masses (1005 hPa), local air masses (1011 hPa), east region air masses (1013 hPa), and north region air masses (1014 hPa).

### 3.2. Human Health Variations Influenced by Diverse Air Mass Sources

**Figure 5:**
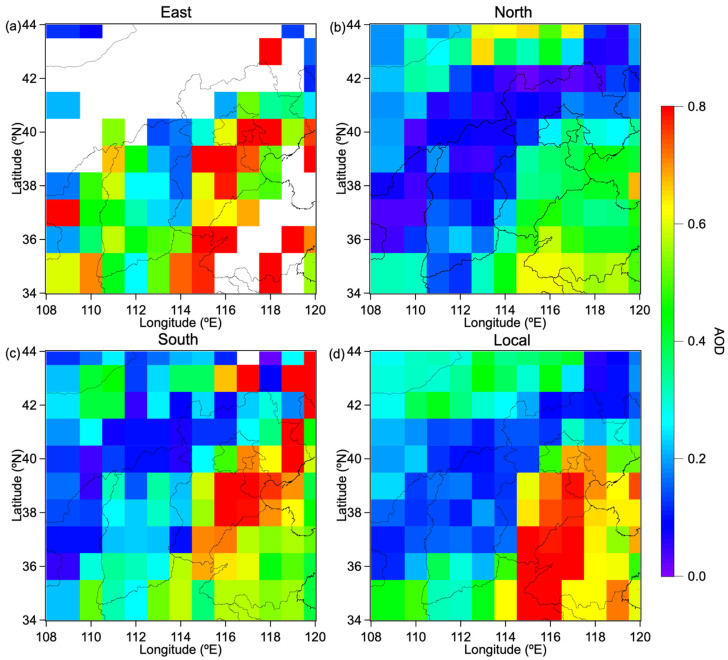
Spatial distribution of mean AOD values by source region: (**a**) eastern, (**b**) northern, (**c**) southern, and (**d**) local air masses during the study period.

MODIS satellite observations of AOD distribution in Beijing and surrounding areas (Figure 5) reveal that AOD values are consistently low when Beijing is influenced by air masses originating from the north, with the maximum AOD in Beijing remaining below 0.3. Under these conditions, even the south region, which is prone to high AOD values, experiences a reduction in AOD to below 0.7. This improvement is primarily attributed to the influence of dry and clean air masses from the north. However, under the influence of air masses from other directions, certain areas in Beijing exhibit higher AOD values, corresponding to the observed PM_2.5_ concentrations. The phenomenon of low AOD in the north and high AOD in the south was also observed in Hu et al.’s research [38]. They investigated the sources of winter pollution events in Beijing at different levels using in situ aircraft measurement instruments, including a passive cavity aerosol spectrometer probe (PCASP) [39] and a single particle soot photometer (SP2, DMT Inc.) combined with weighted potential source contribution function (WPSCF) method [40]. They found that air masses from the south and southwestern regions are more likely to bring polluted aerosols, causing pollution events in Beijing. Comparing Hu et al.’s research on the trend of AOD in mainland China in earlier years [41], it was found that during 2002–2016, the AOD in eastern China, especially in southern Beijing and some southwest areas during the same months as this study period, showed high results, significantly higher than 0.7 and even exceeding 1. However, when compared with our current research results, it is evident that the government’s emission reduction measures have significantly reduced pollution levels.

**Figure 6:**
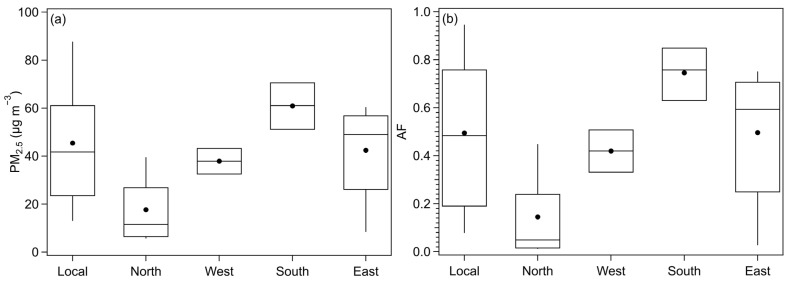
PM_2.5_ (**a**) and AF (**b**) distribution dominated by different air masse sources during the study period. Solid circle indicates mean value, upper and lower boundaries represent 75th and 25th percentiles, and whiskers above and below the box are 90th and 10th percentiles.

Analysis of the impact of different air mass sources on PM_2.5_ concentrations (Figure 6a) in Beijing reveals that the highest average value and concentration distribution occur under the influence of southward air masses, with a mean value of 61 μg m^−3^, falling within the light pollution range stipulated by the Chinese government. This finding is consistent with the research of Hu et al. [38], attributing the increase in PM_2.5_ to the transport of pollutants from high pollution areas in the south region. Air masses from local emissions, eastward transmission, and westward transport also result in mean PM_2.5_ values within the mild pollution range. Hu et al. [13] found that pollution events in Beijing were more influenced by local emissions between 2013 and 2020, which is reflected in our results as high mean and distribution of PM_2.5_ concentrations during local emission periods. Although eastward air masses were previously associated with cleaner conditions, our research indicates that they can now significantly impact pollution levels in Beijing, aligning with Hu et al.’s conclusion that the probability of relatively clean eastward air masses causing pollution has increased after effective control of other incoming pollutants [13]. Air masses from the north remain the primary source of clean air, consistently maintaining average PM_2.5_ levels below 75 μg m^−3^ during the study period.

Figure 6b reveals that the daily attribution scores of PM_2.5_ aerosols from different air mass sources have varying impacts on human health. Due to the higher mean and distribution of PM_2.5_ concentrations in southbound air masses, they pose the greatest risk to human health, with an average AF of 0.75 and a 75th percentile of 0.85. PM_2.5_ from eastward air masses and local emissions also significantly affect human health, with average AF values of 0.5 and 0.49, respectively. Notably, PM_2.5_ from eastward air masses, previously thought to transport relatively clean air, can seriously impact human health, warranting increased attention in future policy adjustments and research. Although the average AF caused by locally emitted PM_2.5_ is slightly lower than that of eastward air masses, the distribution range is wider, suggesting that local emissions may have both high-risk and low-impact effects on human health. Westward air masses have a moderate impact on human health, with a relatively narrow distribution range. Air masses from the north have the least impact on human health, with an average AF of only 0.14, less than one-fifth of the result for southward air masses.

## 4. Conclusions

This study used HYSPLIT trajectory combined with concentration weighted trajectory (CWT) analysis to distinguish the different source directions of air masses in Beijing, and evaluated the distribution of PM_2.5_ in different directions and their impact on human health.

Research has found that, in addition to being influenced by northward air masses, the southern region is the main area where high AOD values occur. This also leads to a higher concentration of PM_2.5_ under southward air mass influence (with an average result of 61 μg m^−3^), which is 1.3 times higher than the average concentration of PM_2.5_ during local emission pollution events, second only to southward air masses. The clean air masses brought by the northward air masses also directly led to the lowest PM_2.5_ concentration in Beijing under the influence of this air mass, with an average result of only 17.7 μg m^−3^. Furthermore, the importance of eastward air masses in the average PM_2.5_ concentration results has increased, which is consistent with the research results of Hu et al. [13] and further indicates that policy adjustments need to pay more attention to the trend areas.

The distribution of AF caused by PM_2.5_ also shows a similar result, with the highest AF value of 0.75 under the influence of southward air masses. The AF value caused by clean air masses under the influence of northward air masses is the lowest, only 0.14, which is less than one-fifth of the result of southward air masses. Under the influence of local emissions, the air mass control with the highest frequency, the mean AF can reach 0.49, but the distribution range is extremely wide. The high value of the 90th percentile is the highest among all incoming air masses, reaching 0.95, while the low value of the 10th percentile can be as low as 0.7. The AF value of the eastward air mass also reached the second highest level among all air masses, further indicating that the proportion of PM_2.5_ pollution caused by the eastward air mass on human health in Beijing is gradually increasing. The higher impact on human health caused by the eastward air mass, emphasizes the need for the Chinese government to prioritize controlling pollutants in the eastern regions. This is crucial due to the profound influence of these pollutants on the health of Beijing residents, and should be considered in future macroeconomic regulations. Consequently, the classification of air masses by source region using our proposed method, along with an evaluation of their health impacts, provides valuable insights for governmental pollution source monitoring and emission reduction policies.

## Data Availability

The datasets used and/or analyzed during the current study are available from the corresponding author upon reasonable request.
